# Surgical Management for Recurrent Bleeding Stercoral Ulcers

**DOI:** 10.7759/cureus.80282

**Published:** 2025-03-09

**Authors:** Amer Mansoor, Richard C Spinale, Bashar Hmoud

**Affiliations:** 1 Surgery, Garden City Hospital, Garden City, USA; 2 General Surgery, Garden City Hospital, Garden City, USA; 3 Gastroenterology, Garden City Hospital, Garden City, USA

**Keywords:** angioembolization, benign rectal ulcer, rectal bleed, recurrent gi bleeding, stercoral ulcer

## Abstract

Stercoral ulcers are a common complication from long-term constipation and generally managed on a case-to-case basis, including options such as endoscopic and surgical interventions. However, recurrent bleeding from rectal stercoral ulcers is exceedingly rare. Being an uncommon cause of long-term GI bleeding, these patients often require rapid resuscitation and hemostasis of the ulcers. We report a case of an elderly woman who presented with progressively deteriorating neurologic function and constipation who subsequently had multiple bouts of recurrent rectal bleeding despite multiple interventions performed by the Gastroenterology and General Surgery teams including endoscopic control with probe cautery and surgical approach with oversewing the vessel. With recurrent bleeding, surgical options, such as bowel resections, can be futile and harmful due to the significant comorbidities often associated with such patients.

## Introduction

Stercoral ulceration is a potential complication from long-term constipation in which fecal impaction results in pressure and eventual bleeding and necrosis in the rectal wall [1]. In the setting of active rectal bleeding, the diagnosis is often made by endoscopy. As previously reported, hemostasis of bleeding rectal ulcers has been successfully achieved through endoscopic and surgical approaches [2]. Endoscopic treatment can involve colonoscopy with probe cauterization, endoclip placement and sclerotherapy injection. Surgical intervention can involve oversewing the bleeding vessel or more aggressive options such as a partial colectomy. However, recurrent episodes of significant bleeding after both of these approaches are a rare incidence. Angiographic embolization of refractory bleeding stercoral ulcers is a novel alternative approach to achieving definitive hemostasis in patient populations with a high morbidity and mortality who may not otherwise tolerate a surgical resection.

## Case presentation

**Figure 1:**
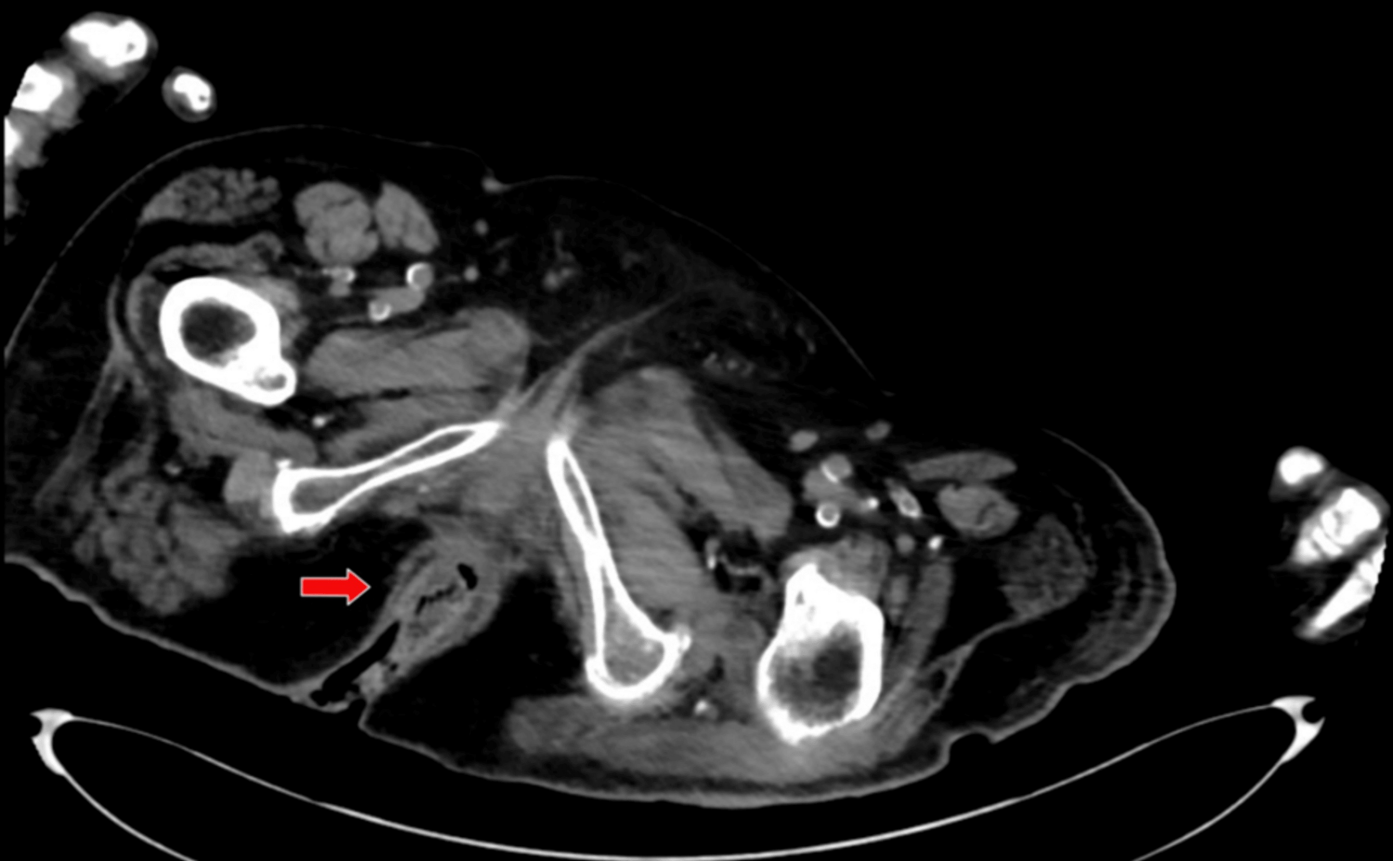
CT abdominal/pelvis view of stercoral colitis showing rectal wall thickening and inflammation

A 72-year-old woman with a significant past medical history of debility, ulcerative colitis, constipation, irritable bowel syndrome, type 2 diabetes, bilateral diabetic foot ulcerations, hypertension, hyperlipidemia presented to the emergency department from her nursing home with a chief complaint of altered mental status and rectal bleeding. Due to the patient's altered mental status and unavailable family contacts, the patient's medical history was mostly obtained from medical records. Per outside chart review, patient has had multiple previous hospitalizations with similar presentations of rectal bleeding dating back to six years prior in which a colonoscopy identified stercoral ulcers and mild ulcerative colitis for which the patient was started on mesalamine. During the patient's hospitalization, the rectal bleeding was described on initial presentation to be bright red and significant in volume with no evidence of melena. Patient was severely anemic with a hemoglobin of 6.1. CT abdomen and pelvis was performed reporting no evidence of active bleeding. CT imaging was performed in an abdominal and pelvic view as shown in Figure 1.

Gastroenterology team was consulted, and a colonoscopy was performed on an emergent basis. As per the operative report, rectal mucosa was inspected and stercoral ulceration with a large vessel was identified in the distal rectum approximately 1 centimeter from the dentate line. Notable bleeding was observed from the visualized inferior hemorrhoidal artery. To obtain hemostasis, sclerotherapy and cautery were performed using 6 cc injection of epinephrine and gold probe cauterization respectively. Gastroenterology noted that if recurrent bleeding occurred, no further endoscopic intervention could be done and recommended surgical intervention. Two days after the procedure, the patient was noted by nursing staff to have fresh bright red rectal bleeding in significant volume. The hemoglobin dropped by several units, from 10 g/dL to 7 g/dL, and the General Surgery team was urgently consulted for concern of active rectal bleeding with blood loss, anemia, and hypotension. The patient was taken for surgical intervention in the operating room. During the procedure, large amounts of frank blood and clots were drained from the rectum and thorough irrigation was performed. Bleeding stercoral ulcers and internal hemorrhoids were noted at the bilateral posterolateral rectal positions. These two sites were ligated with multiple 0 Vicryl suture using a general utility (GU) needle with deep bites taken into the rectal mucosa. Hemostasis was achieved and rectum was reirrigated. To ensure hemostasis, topical thrombin was applied along with injection of 20 cc Xylocaine with epinephrine. A Surgifoam roll dressing and additional kerlix dressing were inserted into the rectum. Patient had no additional rectal bleeding for the next several postoperative days with improving hemoglobin values but did require a transfer to the Intensive Care Unit for hypoventilation and tachypnea. On postoperative day 5, the patient was noted to have new recurrent fresh rectal bleeding along with a downtrending hemoglobin. General Surgery team topically applied hemostatic clotting agents to the patient's sites of rectal bleeding at bedside. Over the next week, the patient continued to have persistent rectal bleeding with downtrending hemoglobin and multiple blood transfusions, and discussions were made to possibly return to the operating room; however, patient remained in a surgically unstable status along with concern that additional efforts at oversewing the same site of bleeding rectal mucosa would be futile. Upon literature review, a few reported cases existed of patients with similar recurrent stercoral ulcer bleeding and the possible indication for an angioembolization. The patient's case was discussed with the Interventional Radiology team regarding performing an angioembolization of the inferior hemorrhoidal artery. However, in the setting of a community-based hospital with limited interventional radiology capabilities, the patient would be unable to have such a procedure performed here. A nearby tertiary care hospital with a wider span of Interventional resources was contacted and the patient was accepted to transfer with anticipation for their Interventional Radiology team to perform an angioembolization of the inferior hemorrhoidal artery. Per chart review, angioembolization was performed of the bleeding artery. Available documentation does not show evidence of rectal bleeding immediately after the procedure. Following the procedure, the patient's family had an extensive conversation with the primary medicine team and elected to make the patient hospice care. Patient was transferred to a hospice facility and no further documentation is available on the patient for recurrence of stercoral ulcer bleeding.

## Discussion

Stercoral ulcers are associated with several different causes including long term hospitalization, chronic constipation and fecal impaction. Pressure from the hardened fecaloma can cause necrosis of the bowel wall; the lesions typically occur in the sigmoid colon and rectum along the antimesenteric margin [3]. The differential diagnosis of a rectal ulcer near the anus can include conditions such as inflammatory bowel disease, lesions developed from a rectal infection and solitary ulcer syndrome. The CT abdomen and pelvis imaging provided detail that the rectal ulceration in this case presentation had an inflamed wall characteristic of stercoral colitis. In addition, the patient's presentation of a known history of chronic constipation, the Gastroenterology team's findings on endoscopy, the General Surgery team's findings in the operating room and no notable findings of infection all guide the diagnosis towards a stercoral ulcer. The occurrence of bleeding from stercoral ulcers is not significantly noted in the literature review. The management for bleeding stercoral ulcers includes resuscitative measures followed by treatment geared towards endoscopic hemostasis, which can include endoscopic multipolar electrocoagulation and injection therapy. Persistent bleeding despite these endoscopic measures has mostly been surgical intervention such as an abdominal rectosigmoid resection or a subtotal colectomy [4]. With the patient demographic most susceptible to recurrent bleeding ulcers being the elderly population, these individuals can have a poor postoperative management and mortality [4]. When suturing the rectal mucosa in an oversewed fashion, the course of the superior and inferior hemorrhoidal arteries should be distinguished. This distinction can be made in the operating room during rectal examination with an understanding of the rectal anatomy and distinguishing the pectinate line. The superior hemorrhoidal artery is a continuation of the inferior mesenteric artery. The artery descends into the pelvic region between the layers of the sigmoid mesentery before crossing the left common iliac artery and vein. The superior hemorrhoidal artery then branches at the level of the third sacral vertebra before running on both sides of the rectum. These branches will divide into smaller branches at about 10 or 12 cm from the anus [5]. The inferior hemorrhoidal artery arises from the internal pudendal artery. The inferior hemorrhoidal artery divides at the pudendal canal into smaller branches, which cross the ischioanal fossa. These branches anastomose with several other arteries including the superior and middle rectal arteries. These anastomoses can be seen during angiography performed for hemorrhoidal artery embolization [5].

Angiographic embolization can be an appropriate intervention to consider for poor surgical candidates. As shown in the literature review, angiographic embolization has successfully controlled lower gastrointestinal hemorrhaging from rectal ulcerations in patients who have prohibitive risks for surgery and who have failed attempts at endoscopic interventions [3]. This case specifically illustrates a patient who had recurrent bleeding despite multiple measures through endoscopic and surgical intervention; the patient only found definitive hemostatic control after being transferred to a tertiary care center with the Interventional Radiology department performing angiographic embolization successfully. A limitation to the case was the patient's family electing to proceed with hospice several weeks after the procedure. This limited the continued surveillance of the patient to evaluate for any potential recurrent bleeding. The case presentation demonstrates both the need to detect potential candidates of angiographic embolization early on and the importance of continued surveillance after this potentially definitive hemostatic and less morbid procedure for recurrent bleeding stercoral ulcerations.

## Conclusions

Sterocoral ulcerations are becoming a more prevalent topic in Gastroenterology and General Surgery with the focus on prevention of fecalomas and management of early complications; however, there is limited literature discussing the approach to a recurrent bleeding ulcer. Endoscopic measures have been used for initial hemostasis and surgical intervention has been outlined for recurrent bleeding, however angiographic embolization offers an alternative route that can be both definitive and less morbid. As more patients start to receive embolization therapy, these patients must continue to have regular surveillance and monitor for any episodes of additional bleeding. In patients with fecal impaction or stercoral colitis, it is important to identify at-risk patients such as those dealing with chronic constipation. Management should include advising patients about proper diet and pharmacologic therapy to help prevent constipation. Osmotic and stimulant laxatives may be considered as a first-line treatment for patients with constipation. This case illustrates the importance of early recognition of recurrent bleeding from a stercoral ulcer and the need to further investigate the benefits of angiographic embolization.

## References

[1] Costouros NG, Niho H, Mahadevan U, Kerlan RK Jr, Bloom AI (2009). Angiographic embolization for control of life-threatening hemorrhage from benign rectal ulcers. J Vasc Interv Radiol.

[2] Hendrickson RJ, Diaz AA, Salloum R, Koniaris LG (2003). Benign rectal ulcer: an underground cause of inpatient lower gastrointestinal bleeding. Surg Endosc.

[3] Matsushita M, Hajiro K, Takakuwa H, Nishio A, Tominaga M (1998). Bleeding stercoral ulcer with visible vessels: effective endoscopic injection therapy without electrocoagulation. Gastrointest Endosc.

[4] Ahmad AI, Sebastian S, Govindu R, Ammar H (2021). It is not diverticular bleeding! A case of stercoral ulcer. Am J Med.

[5] Smith ME, Morton DG, Smith ME, Morton DG (2021). 10 - The colon. The Digestive System (Second Edition).

